# Inpatient Initiation of Cobenfy (Xanomeline-Trospium Chloride) During Acute Psychotic Decompensation in a Patient With Schizoaffective Disorder: A Case Report

**DOI:** 10.7759/cureus.106836

**Published:** 2026-04-11

**Authors:** Preston A Phommathep, Spencer Larson, Scott Zanella, Nina Huang, David Snow

**Affiliations:** 1 Anesthesiology, Lake Erie College of Osteopathic Medicine, Erie, USA; 2 Osteopathic Medicine, Lake Erie College of Osteopathic Medicine, Erie, USA; 3 Psychology, Thomas Jefferson University, Philadelphia, USA; 4 Psychology, Millcreek Community Hospital, Erie, USA

**Keywords:** cobenfy, schizo-affective disorders, treatment-resistant psychosis, treatment resistant schizophrenia, xanomeline-trospium

## Abstract

Xanomeline-trospium chloride (Cobenfy) is a novel muscarinic M1/M4 receptor agonist and a peripherally restricted antimuscarinic combination that represents a non-dopaminergic treatment approach for schizophrenia and related psychotic disorders. Although randomized clinical trials have demonstrated efficacy, published experience with inpatient initiation remains limited, particularly in patients undergoing transition from complex psychotropic polypharmacy. We describe the case of a 25-year-old nonbinary individual with schizoaffective disorder, depressive type, who was admitted for acute worsening of command auditory hallucinations and intermittent suicidal ideation in the setting of persistent psychotic symptoms despite multiple prior medication trials, including clozapine. During hospitalization, a structured medication wash was performed with sequential tapering and discontinuation of multiple psychotropic agents to reduce medication burden and clarify baseline symptomatology. Xanomeline-trospium was subsequently initiated and titrated to a therapeutic dose. Following initiation, the patient demonstrated improvement in the intensity and frequency of psychotic symptoms, resolution of active suicidal ideation by discharge, and good behavioral stability throughout hospitalization. The medication was well tolerated, with no extrapyramidal symptoms or other clinically significant adverse effects. This report highlights the feasibility and short-term tolerability of inpatient xanomeline-trospium initiation during a transition from complex psychotropic polypharmacy in a patient with acute psychotic decompensation.

## Introduction

Schizophrenia spectrum disorders are an umbrella term describing a group of chronic mental illnesses that are primarily characterized by an individual’s detachment from reality, which is called psychosis. Psychosis may manifest as various symptoms such as hallucinations or delusions [1]. Under the Diagnostic and Statistical Manual of Mental Disorders, Fifth Edition, Text Revision (DSM-5-TR) criteria, these disorders are distinguished by symptom profile and duration. Brief psychotic disorder involves at least one core positive psychotic symptom (delusions, hallucinations, or disorganized speech) lasting at least one day but less than one month, with a full return to baseline functioning. Schizophreniform disorder meets similar symptom criteria but persists for one to six months. Schizophrenia is diagnosed when at least two characteristic symptoms are present, including at least one positive symptom, with associated functional decline and continuous signs of illness for at least six months.

Schizoaffective disorder is a condition that combines schizophrenic symptoms and mood disorder symptoms (such as hallucinations combined with depression). Psychiatric decompensation refers to a gradual decline in one’s mental health, leading to a breakdown. This often occurs in individuals who may already be struggling with mental health, and new stressors, traumatic events, or major changes can lead to a complete breakdown. Acute psychotic decompensation is a clinical term used to describe a marked worsening of psychotic symptoms and functional impairment in a patient with an underlying psychiatric illness, often prompting urgent psychiatric evaluation or inpatient stabilization. In this case, the decompensation was evident at the time of admission and was characterized by worsening command auditory hallucinations, impaired reality testing, safety concerns, and progressive functional decline [1].

Acute psychotic decompensation refers to a rapid exacerbation of psychotic symptoms in a patient with an underlying psychiatric disorder, characterized by worsening hallucinations, delusions, disorganization, and/or impaired functioning, often necessitating inpatient stabilization [1]. Schizophrenia-spectrum disorders are commonly treated with dopamine D2 receptor antagonists or partial agonists. Although effective for positive symptoms through modulation of mesolimbic dopaminergic pathways, these agents are frequently associated with adverse effects, including extrapyramidal symptoms due to dopamine D2 receptor blockade in the nigrostriatal pathway, hyperprolactinemia resulting from effects in the tuberoinfundibular pathway, as well as metabolic syndrome and sedation, partly mediated through histaminergic, serotonergic, and other off-target receptor interactions [2,3]. These adverse effects can contribute to poor adherence and long-term morbidity [2,3]. In complex or treatment-resistant cases, patients are often managed with multiple psychotropic medications, increasing the risk of cumulative adverse effects and complicating treatment strategies [4].

Cobenfy (Bristol Myers Squibb, Princeton, NJ) represents a mechanistically distinct approach by using a formulation of xanomeline-trospium chloride [5]. Xanomeline is a centrally acting muscarinic M1 and M4 receptor agonist that modulates cortical and subcortical signaling pathways implicated in psychosis. M1 receptors are highly expressed in the prefrontal cortex and hippocampus and play a critical role in cognitive processing, synaptic plasticity, and glutamatergic transmission [6,7]. Activation of M1 receptors enhances cortical excitatory signaling and may improve deficits in executive function and negative symptoms through modulation of pyramidal neuron activity [6,7].

M4 receptors are expressed in the striatum and modulate dopaminergic neurotransmission through inhibitory effects on dopamine release. Specifically, M4 receptor activation reduces presynaptic dopamine release in the mesolimbic pathway and attenuates dopaminergic signaling within the nucleus accumbens [8]. This indirect modulation of dopamine transmission contrasts with traditional antipsychotics, which directly block D2 receptors. By decreasing dopaminergic tone without receptor blockade, xanomeline reduces positive psychotic symptoms while preserving physiologic dopamine signaling in the nigrostriatal and tuberoinfundibular pathways, thereby minimizing the risk of extrapyramidal symptoms and hyperprolactinemia [9,10]. Additionally, cholinergic modulation through M1 and M4 receptors interacts with glutamatergic and GABAergic circuits implicated in schizophrenia pathophysiology, supporting a network-based model of disease rather than a purely dopaminergic hypothesis [11].

At the same time, trospium chloride is a peripherally restricted antimuscarinic agent designed to mitigate peripheral cholinergic adverse effects without significant central nervous system penetration [9,10]. Its inclusion reduces peripheral cholinergic side effects such as nausea, vomiting, diarrhea, and hypersalivation observed with xanomeline monotherapy [12]. This combination allows indirect modulation of dopaminergic pathways without direct D2 receptor antagonism [9]. Clinical trials, including EMERGENT-2 and EMERGENT-3, have demonstrated statistically significant reductions in Positive and Negative Syndrome Scale (PANSS) scores with xanomeline-trospium chloride compared to placebo, with effect sizes comparable to second-generation antipsychotics [2,12-14]. These studies also demonstrate a tolerability profile characterized primarily by gastrointestinal and autonomic effects rather than extrapyramidal or metabolic adverse effects [2,12-14].

However, there is limited literature describing inpatient initiation in patients undergoing discontinuation of multiple antipsychotic agents. We present a case of a patient with schizoaffective disorder (depressive type), with prior diagnoses including schizoaffective disorder, bipolar type, generalized anxiety disorder, and borderline personality disorder. The patient had an extensive history of medication trials with minimal or no improvement, and most medications were discontinued to initiate xanomeline-trospium. The patient was able to minimize polypharmacy and showed improvement within one week after initiating treatment, with a reduction of the chief complaint of auditory hallucinations.

## Case presentation

Patient information

A 25-year-old nonbinary individual with a history of schizoaffective disorder (depressive type), borderline personality disorder, and generalized anxiety disorder presented with worsening command auditory hallucinations instructing self-harm, somatic passivity phenomena, urinary incontinence, and intermittent suicidal ideation with multiple prior attempts, uncertain in number and date of occurrence; during the admission interview, the patient did not report a plan. The patient described the hallucinations as “voices telling me to hurt myself and saying they can control my body,” which contributed to distress and fear. A command hallucination the patient reported included “[they are] telling me to jump out the window,” exemplifying a second-person command hallucination. The described progressive functional decline led to their eventual admission.

The patient’s psychiatric course was notable for chronic symptom persistence despite multiple medication trials, including both first- and second-generation antipsychotics, mood stabilizers, and adjunctive anxiolytics. They endorsed inconsistent adherence in the past, partly due to perceived lack of efficacy and adverse effects. Clozapine had been initiated previously due to persistent psychotic symptoms despite trials of multiple antipsychotic agents, consistent with treatment-resistant illness. The patient has also had one trial of electroconvulsive therapy (ECT) in the past year, but reported that they had multiple adverse effects, such as extreme tiredness and seizures, preferring to avoid ECT from now on. When offered ECT, the patient would strongly refuse with informed consent and capacity to do so. This pattern suggested a treatment-resistant course, characterized by persistent psychotic symptoms despite adequate pharmacologic exposure.

Psychosocially, the patient had limited social support and ongoing functional impairment, with difficulty maintaining stability outside structured environments. There was no clearly identified acute psychosocial stressor precipitating the current episode, suggesting an endogenous progression of illness rather than reactive decompensation. Family psychiatric history was notable only for the father having schizoaffective disorder. The chronicity and severity of symptoms, along with early onset and functional decline, raised concern for a biologically driven and refractory disease course.

Laboratory evaluation on admission was unremarkable, and no acute medical or toxicologic contributors to the patient’s symptoms were identified. As shown in Table 1, the patient was receiving multiple psychotropic medications with limited clinical benefit.

**Table 1:** Medications the patient was taking at the time of admission PO: by mouth (oral); BID: twice daily; Q6H: every 6 hours; PRN: as needed; AM: morning; PM: night; EPS: extrapyramidal symptoms

Medication	Dose	Schedule	Route	Indication
Haloperidol	10 mg	BID (AM, PM)	PO	Psychosis
Lithium carbonate	600 mg	BID (AM, PM)	PO	Mood stabilization
Mirtazapine	30 mg	1900	PO	Depression/sleep
Desvenlafaxine	50 mg	Daily	PO	Depression
Buspirone	10 mg	BID (AM, PM)	PO	Anxiety
Benztropine	0.5 mg	BID (AM, PM)	PO	EPS prophylaxis
Hydroxyzine pamoate	50 mg	Q6H PRN	PO	Anxiety
Clozapine	200 mg	Daily (AM)	PO	Psychosis
Clozapine	300 mg	Daily (PM)	PO	Psychosis

Complete records of the duration of prior medication trials were not available because the patient had received treatment at facilities outside the treating hospital’s medical record system. The patient had a chronic history of psychotic illness with persistent symptoms over time; however, the exact duration of the acute worsening immediately preceding admission could not be fully established from the available records. At the time of admission, the patient demonstrated an acute exacerbation in symptom severity, characterized by increased intensity of command auditory hallucinations, emergence of safety concerns, and progressive functional decline necessitating inpatient stabilization, consistent with acute-on-chronic psychotic decompensation. This raised the clinical question of whether ongoing symptoms were due to inadequate response to dopamine-based therapies, medication intolerance, or the cumulative effects of polypharmacy. Importantly, this acute deterioration was already present before initiation of the medication wash, supporting the conclusion that the decompensation reflected progression of the underlying psychiatric illness rather than a consequence of medication changes.

Mental status examination

The mental status examination (MSE) is a structured, cross-sectional, open-sourced assessment of a patient’s current psychological functioning based on direct observation and interview, serving as the psychiatric equivalent of the physical exam. It is used to support diagnosis, assess illness severity and safety risk, guide management decisions, and provide a standardized framework for clinical communication and longitudinal comparison [15-17]. Table 2 summarizes the patient’s MSE at the time of admission, providing a systematic evaluation across the key domains.

**Table 2:** Mental status examination upon admission

Domain	Findings	Normal/expected findings
Appearance and behavior	Appears stated age, cooperative, psychomotor retardation present	Appears stated age, well-groomed, cooperative, normal psychomotor activity
Speech	Slow rate, normal volume, spontaneous	Normal rate, rhythm, volume, and spontaneity
Mood and affect	Mood described as “ok,” affect blunted	Mood congruent with situation; affect full range, reactive, and appropriate
Thought process and content	Initially notable for command auditory hallucinations and passivity phenomena, where they believed voices were causing them to fall	Logical, linear, goal-directed thought process; no hallucinations, delusions, or abnormal thought content
Insight and Judgment	Poor	Good insight into condition; judgment intact and appropriate
Cognition	Intact	Alert and oriented ×3–4; intact attention, memory, and executive function
Safety	Intermittent suicidal ideation on admission	No suicidal or homicidal ideation

Table 2 highlights notable abnormalities, including psychomotor retardation, slowed speech, blunted affect, impaired insight and judgment, and the presence of command auditory hallucinations and passivity phenomena. Despite intact cognition, the patient exhibited intermittent suicidal ideation, indicating an elevated safety risk. Overall, these findings are consistent with acute psychiatric decompensation characterized by prominent psychotic features, including worsening auditory hallucinations in which the patient reported voices causing them to be “thrown down,” resulting in falls, tactile hallucinations of bugs crawling on their skin, as well as staff noting pacing behavior and increased confusion, which raised safety concerns on the unit.

The patient also reported believing they were “living in a simulation,” expressed uncertainty about what was real, and described fears that artificial intelligence was controlling people and events around them. This persistent fixation on distinguishing reality from unreality caused marked distress and directly impaired daily functioning, further supporting significant functional decline, a hallmark of psychiatric decompensation according to the DSM-5-TR [1]. The persistence of command auditory hallucinations and passivity phenomena despite extensive pharmacologic treatment further supported a diagnosis of treatment-resistant psychosis.

Treatment course

At the time of admission, the patient was prescribed an extensive psychotropic regimen (Table 1), reflecting multiple prior pharmacologic attempts to manage persistent psychotic symptoms. Despite treatment with several agents, including clozapine, the patient continued to experience severe command auditory hallucinations, including voices telling the patient to jump from high platforms, passivity phenomena, and intermittent suicidal ideation, indicating an inadequate clinical response to the current regimen. This presentation raised concern for treatment-resistant psychosis, particularly in the setting of persistent symptoms despite exposure to multiple antipsychotic medications and substantial baseline polypharmacy, which also increased the potential for adverse effects and pharmacodynamic interactions.

Although the exact duration and adequacy of all prior medication trials could not be fully verified, the patient’s longitudinal treatment history and prior initiation of clozapine suggested a clinical course consistent with treatment-resistant illness. Complete records detailing the duration of prior trials and the rationale for specific medication additions, including clozapine, were not available, likely because the patient had received care across multiple outside facilities not integrated into the treating hospital’s medical record system. It was presumed that clozapine was initiated before admission because of persistent psychotic symptoms despite prior treatment attempts, as it is generally reserved for patients with insufficient response to standard antipsychotic therapy.

Importantly, the patient’s acute psychotic worsening preceded medication simplification and was the reason for admission rather than a consequence of tapering. Given these factors, a decision was made to pursue a structured medication simplification strategy. The goal was to reduce medication burden, minimize potential drug interactions, and better characterize the patient’s baseline symptomatology. A gradual medication wash was initiated (Table 3), with sequential tapering and discontinuation of antipsychotics, mood stabilizers, and adjunctive agents. These adjustments were implemented in a staggered fashion to reduce withdrawal effects and allow for close clinical monitoring. The sequence of medication adjustments and the corresponding clinical rationale during hospitalization are summarized in Table 3.

**Table 3:** Medication timeline and clinical course throughout hospitalization PRN: as needed (orally); Q6H: every 6 hours; AM: morning; PM: evening; BID: twice daily; mg: milligram; IM: intramuscular

Hospital day	Medication change	Clinical course/rationale
Day 1 (admission)	PRN haloperidol 5 mg Q6H initiated; desvenlafaxine discontinued	Worsening agitation and hallucinations; simplification of non-essential medications
Day 6	Clozapine reduced to 200 mg BID; buspirone discontinued	Begin a gradual medication wash to address polypharmacy
Day 8	Mirtazapine decreased to 15 mg PM	Reducing sedating agents during wash
Day 10	Lithium decreased to 300 mg BID	Tapering mood stabilizer
Day 13	Clozapine reduced (150 mg)	Continued antipsychotic taper
Day 15	Lithium AM dose discontinued	Progressive lithium taper
Day 16	Lithium fully discontinued; mirtazapine discontinued	Further simplification of the regimen
Day 21	Clozapine further reduced to 100 mg (final taper phase); PRN haloperidol discontinued; ziprasidone 20 mg IM BID PRN started	Transition away from prior antipsychotics; PRN alternative added
Day 23	Scheduled haloperidol reduced to 5 mg BID	Continued antipsychotic taper
Day 27	Clozapine discontinued; topiramate discontinued; desmopressin reduced	Final stages of medication wash
Day 31	Cobenfy (xanomeline–trospium chloride) initiated at 50 mg BID	Initiation of novel non-dopaminergic therapy
Day 33	Doxepin 10 mg PM started	Management of insomnia
Day 35	Cobenfy increased to 100 mg BID	Reached the target therapeutic dose
Day 38 (Discharge)	No further medication changes	Improvement in hallucinations and mood; resolution of suicidal ideation

Despite progressive reduction of multiple psychotropic medications, the patient continued to exhibit persistent psychotic symptoms, including command hallucinations and disorganization, further supporting a treatment-resistant disease course rather than medication-induced or polypharmacy-related symptomatology. Given the patient’s limited response to prior dopamine-based therapies and their informed refusal of further ECT due to prior adverse effects and limited perceived benefit, a decision was made to initiate Cobenfy, targeting cholinergic pathways as a mechanistically distinct approach. The medication was initiated on hospital day 31 at 50 mg/10 mg twice daily, following partial completion of the medication wash, and was subsequently titrated to 100 mg/20 mg twice daily after four days, and this dose was maintained through discharge.

Throughout hospitalization, the patient remained behaviorally stable, requiring no seclusion or restraints. Vital signs remained within normal limits, and no extrapyramidal symptoms were observed, consistent with the medication’s non-dopaminergic mechanism of action [2,12-14]. Supportive medications included doxepin 10 mg nightly for insomnia, along with a bowel regimen, vitamin D supplementation, and atropine drops as needed for sialorrhea.

Outcome

Over the course of hospitalization, the patient demonstrated gradual clinical improvement following the structured medication wash and initiation of xanomeline-trospium. Initially presenting with persistent command auditory hallucinations and intermittent suicidal ideation, the patient reported a reduction in the intensity and frequency of hallucinations after initiation and titration of therapy. By the time of discharge, the patient no longer endorsed active suicidal ideation and demonstrated improved engagement with the treatment milieu.

The patient tolerated xanomeline-trospium at a dose of 100 mg/20 mg twice daily without the development of extrapyramidal symptoms or clinically significant adverse effects. Vital signs remained stable, and no episodes requiring seclusion or restraints occurred during hospitalization. A plan for close outpatient psychiatric follow-up was established at discharge to monitor long-term response, adherence, and tolerability. The observed improvement following initiation of xanomeline-trospium, after failure of multiple prior pharmacologic trials, further supports its potential role in treatment-resistant psychosis.

## Discussion

This case highlights the feasibility of initiating Cobenfy in an inpatient setting for acute psychotic decompensation in a patient with schizoaffective disorder and significant baseline polypharmacy. The patient’s presentation, characterized by persistent psychotic symptoms despite treatment with clozapine, adjunctive agents, and prior ECT, is consistent with treatment-resistant illness, which often necessitates complex medication regimens and increases the risk of adverse effects and drug interactions. As described in the case presentation, the patient had previously undergone ECT but experienced significant adverse effects, including profound fatigue and reported seizure-related complications, and subsequently declined further treatment with demonstrated decision-making capacity. Although ECT remains an established intervention for refractory mood and psychotic disorders, its limited tolerability and perceived lack of sustained benefit in this case reduced its feasibility as an ongoing therapeutic option.

In the context of inadequate response to multiple dopamine-based pharmacologic agents and the patient’s refusal of further ECT, a non-dopaminergic treatment approach was pursued. Initiation of Cobenfy, therefore, represented a rational next step, targeting a mechanistically distinct neurochemical pathway. A structured and gradual medication wash was implemented to reduce polypharmacy and clarify treatment response (Table 3). This approach allowed for careful monitoring of withdrawal effects and psychiatric stability while transitioning away from multiple dopamine-targeting agents. Notably, Cobenfy was initiated before complete discontinuation of all prior medications, facilitating a smoother pharmacologic transition and reducing the risk of acute decompensation.

The patient’s treatment resistance is likely multifactorial. Despite adequate trials of multiple antipsychotic agents, including clozapine, symptoms persisted, suggesting a biologically refractory disease course. Contributing factors may include illness chronicity, comorbid borderline personality disorder affecting emotional regulation and symptom perception, and prior inconsistent adherence, which may have limited sustained therapeutic response. Additionally, prolonged exposure to multiple psychotropic agents may have contributed to pharmacodynamic tolerance or diminished responsiveness to dopamine-mediated therapies. These factors collectively support a model of treatment resistance extending beyond simple medication failure and highlight the need for alternative mechanistic approaches. This reinforces the emerging concept that treatment-resistant psychosis may involve neurobiological mechanisms beyond dopaminergic dysregulation alone.

Cobenfy represents a novel, non-dopaminergic treatment strategy targeting muscarinic M1 and M4 receptors, in contrast to traditional antipsychotics that primarily act via dopamine D2 receptor antagonism or partial agonism. This distinction is clinically relevant, as shown in Table 4: D2 receptor blockade is associated with well-established adverse effects, including extrapyramidal symptoms via the nigrostriatal pathway, hyperprolactinemia via the tubuloinfundibular pathway, and metabolic complications related to off-target receptor activity [2,12-14]. In contrast, clinical trials of Cobenfy have demonstrated efficacy in reducing psychotic symptoms with a tolerability profile characterized primarily by cholinergic and anticholinergic effects, such as gastrointestinal symptoms and mild autonomic changes, with low rates of extrapyramidal symptoms [3,9-10]. This contrast is summarized in Table 4.

**Table 4:** Comparison of Cobenfy mechanisms and adverse effects with conventional (atypical) antipsychotics used in schizophrenia EPS: extrapyramidal symptoms

Feature	Cobenfy (xanomeline–trospium chloride)	Conventional (atypical) antipsychotics
Mechanism	M1/M4 agonism	Dopamine (D_2_) antagonism, Serotonin receptor (5HT_2A_) antagonism, Histamine (H_1_) antagonism, α_1_ antagonism
Extrapyramidal symptoms	Minimal	Moderate to high
Prolactin elevation	Minimal	Common
Metabolic effects	Minimal	Significant
Common adverse effects	Gastrointestinal, mild autonomic	EPS, sedation, metabolic syndrome
Cognitive effects	Potential improvement	Often neutral or impairing

In this case, the patient tolerated treatment without extrapyramidal symptoms or significant autonomic instability, consistent with prior studies. The absence of dopamine-related adverse effects may be particularly advantageous in patients with prior intolerance to antipsychotics or those at risk for movement disorders or metabolic complications. Additionally, reducing a complex medication regimen to a more streamlined approach may improve long-term adherence and reduce the cumulative side-effect burden. This case also underscores the importance of addressing prescribing cascades in psychiatric care. Several medications in the patient’s initial regimen were likely prescribed to manage side effects of primary psychotropic agents, contributing to overall treatment complexity. The inpatient setting provided an opportunity to safely deprescribe and reassess the necessity of each agent.

The limitations of this report include the single-patient design and the inability to establish causality between Cobenfy initiation and clinical improvement. Additionally, long-term outcomes remain unknown as follow-up data beyond discharge were unavailable. Further studies, including real-world observational data and head-to-head comparisons with standard antipsychotics, are needed to better define the role of Cobenfy in the treatment of schizophrenia-spectrum disorders.

## Conclusions

In this case of schizoaffective disorder with acute-on-chronic psychotic worsening present at admission, inpatient initiation of Cobenfy following a structured medication wash was feasible and well tolerated. The patient’s acute decompensation preceded medication simplification, supporting the conclusion that the presenting psychotic exacerbation reflected the underlying illness rather than a consequence of the wash itself. During hospitalization, transition from a complex dopamine-based polypharmacy regimen to a non-dopaminergic treatment approach was associated with improvement in psychotic symptoms and resolution of suicidal ideation without the emergence of extrapyramidal adverse effects. These findings support the potential role of Cobenfy as an alternative treatment strategy in complex patients with persistent psychotic symptoms or limited response to traditional antipsychotics. Further investigation is warranted to evaluate long-term efficacy, safety, and optimal strategies for transitioning from existing antipsychotic regimens.
